# SHRINKING WORLDS: SOCIAL NETWORK OVERLAP FOR OLDER ADULTS WITH AND WITHOUT ADRD AND THEIR CLOSE TIES

**DOI:** 10.1093/geroni/igae098.4352

**Published:** 2024-12-31

**Authors:** Lucas Hamilton, Brea Perry

**Affiliations:** Augustana University, Sioux Falls, South Dakota, United States; Indiana University, Bloomington, Indiana, United States

## Abstract

Alzheimer’s disease and related dementias (ADRD) are an urgent public health issue with psychosocial consequences that reverberate through family systems, weakening key social bonds for diagnosed individuals and caregivers. Feelings of social isolation are associated with worse clinical outcomes for people with ADRD and elevated dementia risk in caregivers. Data from 124 focal-informant dyads were used to examine the characteristics and consequences of shared ties between older adults and their informants – typically a spouse or child. Focal participants were diagnosed as cognitively normal (n = 73) or experiencing ADRD-related cognitive impairment (n = 51). All participants completed separate social network interviews, naming people with whom they discuss important or health-related matters. Using multilevel logistic regression, we found that family members and individuals offering psychosocial support were significantly more likely to be shared ties (all odds ratios > 1.26, all p-values <.05). People who provided instrumental support were more likely to be shared ties only in the networks of ADRD informants (OR = 2.11, p <.01), suggesting they may mediate caregiving tasks on behalf of the person with ADRD. More network overlap was associated (p <.05) with worse general cognition and episodic memory (β = -.261; β = -.323, respectively) among ADRD focals, indicating that loss of social independence is associated with poorer cognition (or vice versa). Higher overlap was also associated with greater loneliness in ADRD informants (β =.282, p =.05), suggesting that lacking social ties outside the caregiving system may threaten caregiver wellbeing.

